# Plant-based and cell-based approaches to meat production

**DOI:** 10.1038/s41467-020-20061-y

**Published:** 2020-12-08

**Authors:** Natalie R. Rubio, Ning Xiang, David L. Kaplan

**Affiliations:** Department of Biomedical Engineering, Tufts University, 4 Colby St., Medford, 02155 Massachusetts USA

**Keywords:** Synthetic biology, Tissue engineering, Environmental impact, Sustainability

## Abstract

Advances in farming technology and intensification of animal agriculture increase the cost-efficiency and production volume of meat. Thus, in developed nations, meat is relatively inexpensive and accessible. While beneficial for consumer satisfaction, intensive meat production inflicts negative externalities on public health, the environment and animal welfare. In response, groups within academia and industry are working to improve the sensory characteristics of plant-based meat and pursuing nascent approaches through cellular agriculture methodology (i.e., cell-based meat). Here we detail the benefits and challenges of plant-based and cell-based meat alternatives with regard to production efficiency, product characteristics and impact categories.

## Abstract (web summary)

Large-scale meat production can have negative impacts on public health, the environment and animal welfare. In this Review, the authors consider plant-based and cell-based approaches to meat production and the challenges they face.

## Introduction

Global production and consumption of meat continue to surge as demand is driven upward by population growth, individual economic gain, and urbanization^1,2^. In 2012, the Food and Agriculture Organization (FAO) of the United Nations projected the global demand for meat would reach 455 M metric tons by 2050 (a 76% increase from 2005)^3^. Likewise, the global demand for fish is projected to reach 140 M metric tons by 2050^4^. The majority of this incline is attributed to middle-income countries (e.g., China), as consumption in higher-income countries is relatively stagnant or marginally decreasing (e.g., United Kingdom) and in lower-income countries, the rate of consumption is fairly constant (e.g., India)^1^. This pattern is consistent with a proposed theory that the relationship between meat consumption and income follows an “inverted U-shaped” trend; consumption initially increases with rises in income but eventually reaches a turning point at which consumption stagnates or declines^5^. This observation may be rationalized by correlations between high income and increased concern for the consequences of animal agriculture^5^.

This rising demand is problematic as current methods of large-scale animal husbandry are linked to public health complications, environmental degradation and animal welfare concerns. With regard to human health, the animal agriculture industry is interconnected with foodborne illness, diet-related disease, antibiotic resistance, and infectious disease^6,7^. Notably, zoonotic diseases (e.g., Nipah virus, influenza A) are linked to agricultural intensification and meatpacking plants in the United States were hotspots for COVID-19 outbreaks^7,8^. Animal agriculture also contributes to environmental issues including greenhouse gas emissions, land use, and water use^1^. The United Nations Intergovernmental Panel on Climate Change released a 2018 report asserting that greenhouse gas emissions must be reduced 45% by 2030 to prevent global temperatures from increasing 1.5 °C; a target that could mitigate catastrophes associated with a 2.0 °C increase^9^. Conventional mitigation techniques include improvements in reforestation, soil conservation, waste management as well as tax policy, subsidies, and zoning regulations^10^. While these strategies remain important, the urgency of climate change may require more transformative approaches. Lastly, with regard to animal welfare concerns, each year billions of animals are killed or suffer either directly (e.g., farm animal slaughter, seafood fishing) or indirectly (e.g., fishing by-catch, wildlife decline due to habitat destruction) in relation to human food systems^11,12^.

The majority of the aforementioned issues can be attributed to the fact that the raw material inputs (i.e., animals) for conventional meat production are inherently unsanitary, inefficient, and sentient. By removing animals from the manufacturing process, several externalities may be alleviated. Plant-based meat (PBM) and cell-based meat (CBM) approaches offer to generate food from non-animal sources. While traditional PBMs (e.g., tofu) have existed for centuries, novel PBM alternatives with enhanced sensory characteristics have been commercialized more recently (Fig. 1)^13^. Other groups have initiated the development of a new field—cellular agriculture. Cellular agriculture describes the tactic of producing commodities from cells, rather than whole organisms or animals, such as CBM; meat grown from muscle or fat cells rather than cows, pigs or chickens. While there is evidence to support certain benefits of these approaches compared to animal-based meat (ABM), it is important to more comprehensively evaluate impacts on human health and the environment as production systems evolve. In addition, widespread adoption of these products will require more direct benefits to the consumer; such as taste, cost, and convenience^14^. This review serves to compare plant-based (i.e., meat analogs composed of plant proteins) and cell-based (i.e., meat analogs generated from cell cultures) methods to educate stakeholders about the strengths and challenges of each approach and highlight areas of uncertainty.

**Figure:**
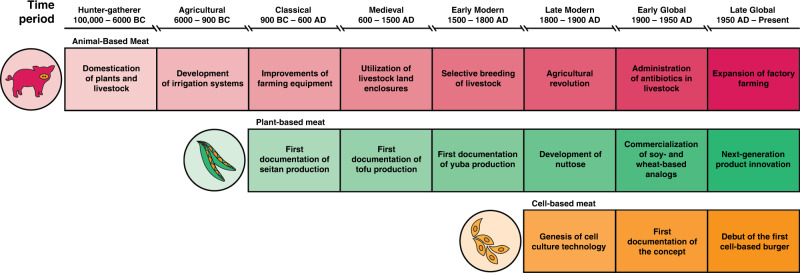
Fig. 1. The history and evolution of animal-, plant- and cell-based approaches to meat production. ^13,87–93^. Humans have consumed plant-based meat (2555 years ago) for only 0.098% of the time period for which their ancestors have consumed animal-based meat (2,600,000 years ago). Likewise, humans have eaten cell-based meat (7 years ago) for only 0.274% of the time period for which they have consumed plant-based meat.

## History and approach

**Fig. 2:**
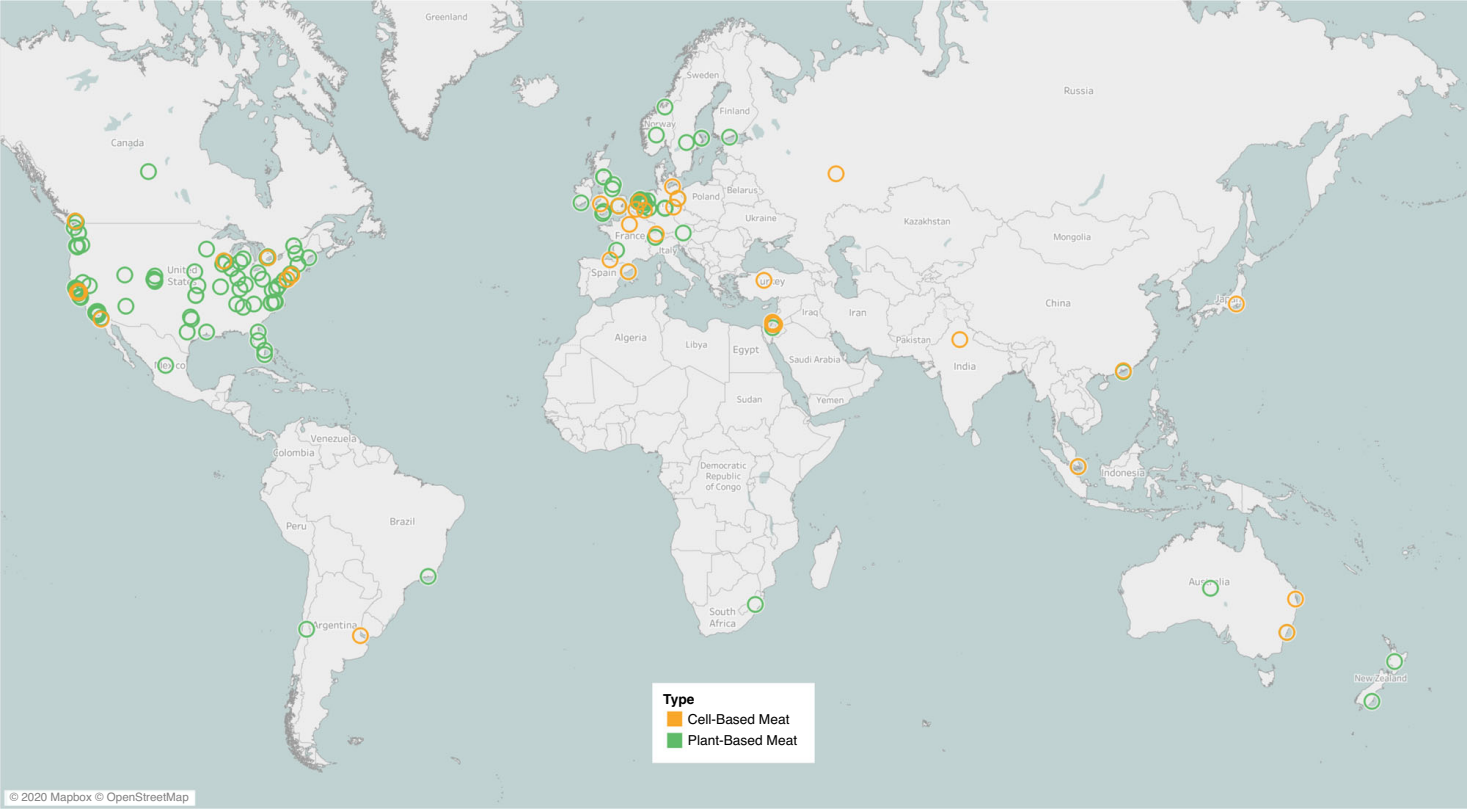
Geographical distribution of plant-based (green circles) and cell-based (orange circles) meat companies. Companies were included as listed in the Good Food Institute alternative protein company database (August 2020).

Plant- and fungi-based meat (PBM) products encompass the flavor, texture, and/or nutritional aspects of meat but are different in composition; namely are made from non-animal sourced materials. Based on the time of development and technical complexity, PBM products can be differentiated into two flexible categories: traditional and novel (i.e., next-generation)^13^. Traditional meat analogs were developed thousands of years ago in Asia and include relatively simple derivatives from soybeans (i.e., tofu, tempeh) or wheat (i.e., seitan)^13^. In contrast, novel PBMs are characterized by the design and marketing of products as near equivalent replacements for ABM with regard to taste, texture, and nutrition. Product categories can also exist between traditional and novel, as they may meet some but not all of the aforementioned criteria. A distribution map of global companies and brands developing novel PBMs can be found in Fig. 2. Typically, the production of PBM includes three steps^15^: (i) Protein isolation and functionalization—Target plant proteins are extracted from plants, some of which are subjected to hydrolysis in order to improve their functionalities such as solubility and cross-linking capacity; (ii) Formulation—The plant proteins are mixed with ingredients to develop meat texture such as food adhesives, plant-based fat and flour. Nutrients are added to match or exceed the nutrient profile of the meat. (iii) Processing—The mixture of plant proteins and other ingredients undergo protein reshaping processes (e.g., stretching, kneading, trimming, pressing, folding, extrusion, etc.) to form a meat-like texture. Innovative technologies being utilized to improve the organoleptic properties of PBMs include shear cell technology, mycelium cultivation, 3D printing, and recombinant protein additives^16,17^.

CBM, also referred to as in vitro meat, lab-grown meat or cultured meat, is meat produced by cultivating cells as opposed to farming animals. CBM technology is based on advances in stem cell biology (e.g., induced pluripotent stem cells) and tissue engineering (e.g., in vitro skeletal muscle grafts) originally purposed for medical applications. CBM production involves four central components: (1) muscle and fat cell isolation and culture, (2) xeno-free culture medium formulation, (3) scaffold development, and (4) bioreactor design; the details of which are described extensively elsewhere^14^. Interestingly, the concept of CBM can be traced back to 1930 when Frederick Smith, the British Secretary for India, envisioned the genesis of “self-reproducing steaks” through an excerpt of his essay collection *The World in 2030 AD*, which reads: “It will no longer be necessary to go to the extravagant length of rearing a bullock in order to eat its steak. From one ‘parent’ steam of choice tenderness, it will be possible to grow as large and as juicy a steak as can be desired^18^.” While CBM has yet to be commercialized in 2020, noteworthy progress has taken place over the past couple of decades. Key milestones include the first CBM patent filed by Willem van Eelen in 1999^19^, the first peer-reviewed research on cultured fish funded by NASA in 2002^20^ and the first cultured beef burger debuted by Maastricht University in 2013. Today, there are dozens of start-up companies around the globe working to bring CBM products to market.

## Economics

Key sources of protein inputs for novel PBM are relatively inexpensive. The majority of plant-based products are primarily formulated with pea, soy or wheat protein. The agricultural prices (received by farmers in the United States) for these key proteins are 3.8–12.7 times lower than prices received for cattle, hogs, and broilers. In fact, sometimes soy and wheat are combined with ABM to reduce costs for processed meat products^21^. When standardized by cost (2009 data) per gram of protein, soybeans ($0.01/g) and wheat ($0.03) are still remarkably less costly than cows ($0.32/g), pigs ($0.22/g), and chickens ($0.12/g)^22^. Despite this glaring discrepancy on the key protein input level, novel PBMs tend to cost more than their animal-based counterparts in a retail setting. This discrepancy may be partially due to processing costs as 94.3% of retail costs for crop products are associated with post-harvest processes, while this accounts for ~50% of the retail costs for beef^22^. Furthermore, aside from primary proteins, PBMs often include plant-based fats, flavor enhancers, and color additives which contribute to cost. Some consumers are willing to pay premiums for meat substitutes linked to personal health benefits^23^.

Economic feasibility is a significant hindrance to CBM commercialization^24^. The cultured beef burger cultivated by Maastricht University in 2013 is reported to have cost $280,400 ($2,470,000/kg) to produce. The production process involved three researchers using bench-scale techniques to culture 20,000 muscle fibers over three months and served as a proof-of-concept rather than an attempt to scale production. A few groups have performed preliminary economic analyses to project the cost of CBM for large-scale production scenarios (Table 1). In 2008, The In Vitro Meat Consortium estimated, by modeling capital and growth medium costs based on data for single-cell protein production, CBM could cost approximately twice as much as chicken^25^. In 2014, a study speculating on the technical, societal, and economic factors of village-scale CBM production calculated a cost range of $11–520/kg dependent on the price of growth medium^24^. Invertebrate (e.g., insect, crustacean) cell culture may present a more cost-efficient platform for CBM production based on the unique properties of insect cells (e.g., xeno-free growth medium, high-density suspension culture)^26,27^. Select companies are targeting high-value products (e.g., foie gras, bluefin tuna, kangaroo meat) in order to lower the bar for reaching price parity. Interesting, a recent consumer acceptance study from the Netherlands reported 58% of participants were willing to pay a 37% premium for cell-based beef compared to conventional animal-based beef^28^.

**Table 1:** Results from preliminary economic analyses of CBM production^24,25^.

Reference	[Doyle et al.,^25^]^a^	[van der Weele & Tramper^24^]^b^
Growth medium-cost range (USD/L)	10.29–11.76	1.33–66.50
CBM cost range (USD/kg)	4.81–5.10	10.39–519.53
CBM per volume growth medium (kg/MT)	193	128

^a^Estimates are based on capital, variable media, and overhead costs for a plant capacity of 15,000 tons/year. The lower limit is estimated for cells grown in suspension and the upper limit is estimated for cells grown in a 3D matrix.

^**b**^Estimates are based on batch production of 2560 kg CBM per batch assuming 20,000 L/batch. CBM cost only considers contributions from growth medium.

## Regulatory framework

PBMs are regulated in a similar manner as other non-animal foods. In the United States, the Food and Drug Administration (FDA), and specifically the Center for Food Safety and Applied Nutrition (CFSAN), oversees food inspection, labeling, packaging, imports, and facility safety. Most PBM products contain simple ingredients that have previously been approved for human consumption. Novel ingredients may be subject to additional evaluation processes. For example, soy leghemoglobin, produced via genetic engineering, filed for “generally recognized as safe” status with the FDA for use as a color additive^17^. In the European Union (EU), current policy and regulation are supportive for alternative proteins innovation and investment. In 2018, the European Commission presented a “EU Protein Plan”, which encourages the production of alternative proteins for human consumption, and listed existing EU policy instruments that “provide options for strengthening the development of EU-grown plant proteins”. Many novel PBM products are classified under the Novel Food Regulation which regulates “food that had not been consumed or do not exist in the EU before 15 May 1997”^23^. Australia, Canada, and New Zealand have also introduced legislation to guide oversight of novel foods^13^. Government oversight is also required for food labeling. In 2018, The United States Cattlemen’s Association petitioned the Food Safety and Inspection Service (FSIS) “to exclude products not derived directly from animals raised and slaughtered from the definition of “beef” and “meat”^29^. The use of terms such as steak, sausage, bacon, fillet, etc. for PBMs is subject to scrutiny and restriction in many EU member states as well.

Oversight for CBM involves the regulation and monitoring of production, packaging, labeling, and marketing. In the United States, CBM will be jointly regulated by the FDA and the United States Department of Agriculture (USDA) based on a decision announced by the departments in 2019^30^. The FDA will regulate cell isolation, storage, growth, and maturation. After cells and tissues have been harvested, the USDA will monitor products through the remainder of the commercialization process and oversee labeling^30^. Scaffold materials may fall under FDA food additive provisions^31^. Even with a joint effort, it will be important to utilize current systems but also to implement new regulation procedures as the technology continues to advance^32,33^. Further complication could arise if companies intend to sell products containing genetically modified (GM) cells. While the USDA regulates GM crops, a FDA New Animal Drugs Application provision views DNA manipulation to fall under the definition of a drug and dictates FDA oversight of GM animals; this could potentially be interpreted to also apply to GM cells^33^. A second concern about regulations is with respect to accurate labeling. Similar to the PBM labeling debate, there is an effort to prevent cell-based products from being labeled as “meat”^29^. Based on the Federal Meat Inspection Act which refers to meat as “any product… made wholly or in part from any meat or portion of the carcass”, there may be justification for CBM to retain its wording. In fact, the North American Meat Institute states that cell-based products likely fall into the definitions of either “meat” or “meat byproduct”^34^. For Europe, CBM could be applicable to the European Union Novel Food Regulation pathway. While the Food Safety Authority has approved GM food production, contingent on thorough safety assessments, many European countries (e.g., France, Germany, Greece) have banned the production and sale of GM foods^35^.

## Organoleptic properties

**Fig. 3:**
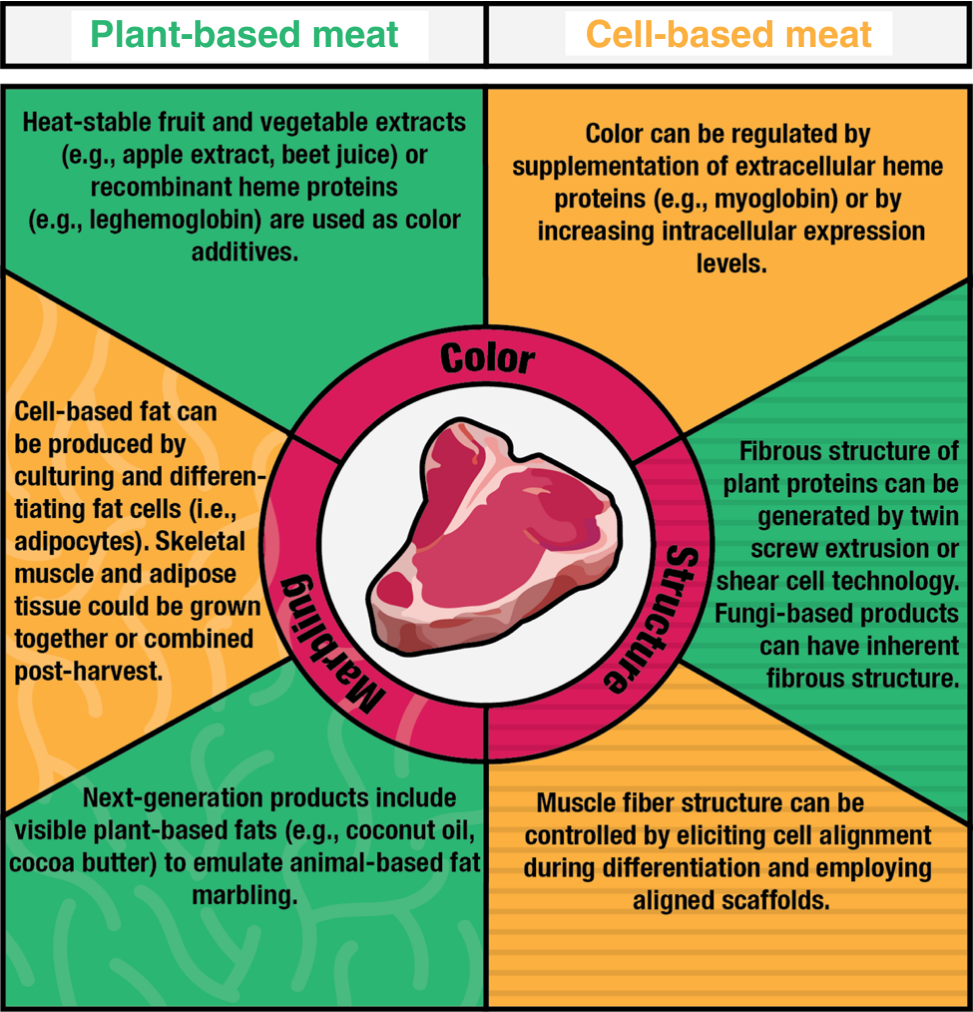
Plant-based and cell-based strategies for emulating appearance properties (color, marbling, structure) of meat. Structure and marbling are significant contributors to the texture of meat as well as appearance.

The chief organoleptic (i.e., sensory) properties of meat are appearance, aroma, flavor (Table 2), and texture. Novel PBMs mimic the look of ABM by manipulating color, fat marbling and structure (Fig. 3). Depending on the product, PBMs aim to emulate the appearance of raw (e.g., ground meat) or pre-cooked meat (e.g., deli slices). Heat-stable fruit and vegetable extracts (e.g., apple extract, beet juice) or recombinant heme proteins (e.g., LegH) are used to both recreate the color of fresh meat and change to brown upon cooking^17,36^. To mimic the appearance of fat, some novel PBM products exhibit visible semi-solid plant-based fats (e.g., coconut oil, cocoa butter). Engineering flavor and aroma profiles are important to recapitulate the taste and smell of meat. In meat analogs, flavor additives are incorporated to add, enhance or mask specific flavor notes and generally compose 3–10% of the product^21^. Many plant proteins are associated with bitter and astringent tastes, which require selective compound removal by post-processing^15^. In particular, soy products have strong grassy, beany and bitter flavors linked to lipoxygenase, saponin and isoflavone compounds which can be reduced through germination or heating^15^. A synthetic meat flavor developed in the 1980s was composed of sugar, amino acids, nucleotides, glycoprotein, monosodium glutamate, salt, and fat and determined by a sensory panel to be equal or superior to meat extract^37^. Recombinant protein additives like LegH can contribute to the flavor as well as the color of PBMs^17^. PBM texture can be influenced by high-moisture extrusion, shear cell technology, mycelium cultivation and 3D printing. Extrusion, shear cell technology and 3D printing rely on applying mechanical, thermal and shear stresses to a protein mixture to obtain a semi-solid fibrous structure^16^. While many strategies are available to engineer and tune the texture of plant proteins, it can be difficult to balance processing methods to achieve desired mechanical properties while also retaining nutritional value^15^. Conversely, mycelium cultivation involves growing filamentous fungi; particular strains of which emulate the microstructure of meat^38^. Quorn™ is a fungal-based PBM that has provided alternatives for chicken nuggets, meatballs, and minced meat since the 1960s^38^. New start-ups (e.g., AtLast Food Co., Emergy Foods) are growing mycelia with goals of generating higher quality cuts of meat, such as steak.

**Table 2:** Precursors and compounds attributable to the aroma and flavor of meat.

Flavor precursor categories^82^	Specific examples^83,84^	Thermal reaction^82^	Flavoring compounds^84–86^	Associated flavor^84–86^
Sugars nucleotides Amino Acids Peptides	Glucose 5′-Adenosine Monophosphate Cysteine Anserine	Maillard Reaction	Pyrazines, Alkylpyrazines, Alkylpyridines, Acylpyridines, Pyrroles, Furans, Furanones, Pyranones, Oxazoles, Thiophenes	Roasted, Caramel, Meaty
Lipids fatty acids	Phospholipids Linoleic acid	Oxidation	Hydrocarbons, Alcohols, Aldehydes, Ketones, 2-Alkylfurans	Fatty, Buttery, Sweet
Vitamins	Thiamine	Degradation	Thiols, Aliphatic Sulfur Compounds, Furans, Thiophenes	Ethereal, Heated Onion, Sulfury

To increase the likelihood of mainstream consumer adoption, CBM must be equivalent or superior to ABM from a sensory perspective^39^. The 2013 cultured beef burger (which contained cultured skeletal muscle tissue but not adipose tissue and was flavored with beet juice, bread crumbs, caramel, egg powder, salt and saffron) was described as tasting “like a real burger” by one panelist and “close to meat, but not that juicy” by another^40^. Since this milestone, more effort has been focused on generating cell-based adipose (i.e., fat) tissue; given its significant contribution to taste and texture. Advances in engineering fat tissue for use in food have been reviewed in depth elsewhere^41^. Aside from skeletal muscle and adipose tissue, ABM also contains connective tissue, vasculature networks, and supporting cell types (e.g., fibroblasts). The discrepancies in complexity may result in nuanced flavor differences between ABM and CBM. CBM experts indicate that key flavor profiles can be achieved by co-cultures, medium supplementation, and/or genetic modification^14,39^. For instance, researchers have explored the effects of supplementing CBM with extracellular heme proteins (e.g., myoglobin)^42^. Myoglobin is associated with the “bloody” flavor of meat and supplementation was observed to improve the color of CBM constructs without impeding muscle cell growth rates^42^. Early cell-based prototypes emulate processed meat (e.g., burgers, sausages, nuggets) as it is more difficult to emulate the appearance and texture of whole cuts of meat (e.g., steak). As researchers in the field begin to focus on textural properties, significant effort will be required to evaluate myriad factors (e.g., cell to scaffold ratio, the impact of cooking, packaging, storage, and shipping) on tissue structure. CBM texture can be influenced both by cultured cells and supportive scaffolding materials. In vitro skeletal muscle tissue can be engineered to emulate the structure of meat by employing differentiation and cell alignment strategies. For example, mechanical tension, electrical stimulation, and/or micropatterned substrates can be employed to induce cell alignment in vitro^43^. A recent study focused on CBM composed of bovine cells coupled with a textured soy protein scaffold; finding some of the samples exhibited texture (i.e., ultimate tensile strength) properties similar to those of native bovine muscle^44^. In addition, a sensory panel tasted the CBM samples and described “a pleasant meaty flavor” and “a typical meat bite and texture”^44^.

## Nutrition

**Fig. 4:**
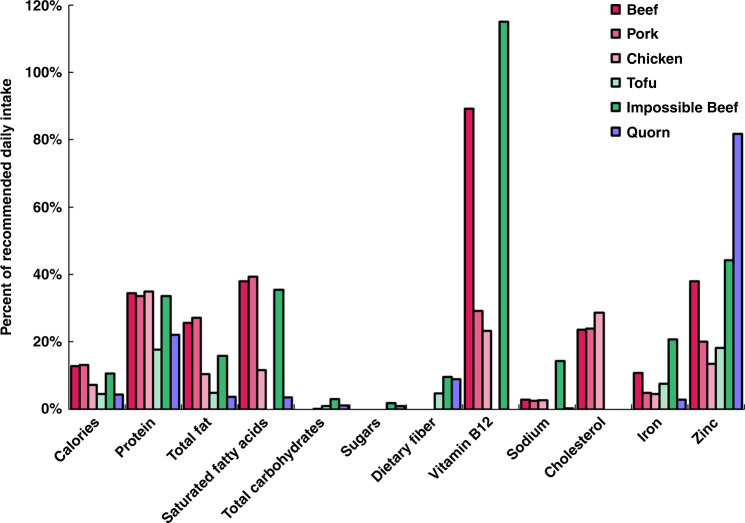
Nutritional value of ABM (beef, pork, and chicken), traditional PBM (tofu), novel PBM (Impossible™ Beef), and mycoprotein (Quorn™) per 100 g wet weight, raw. Nutritional data for ABM and tofu were obtained from the FoodData Central database (FDC ID: 174036, 167902, 171116, 388713) and Impossible™ and Quorn™ data were obtained from company websites. Content is quantified by the percent of recommended daily intake as determined by the FDA^94^.

The key plant-based proteins utilized in PBM formulations (e.g., pea, soy, wheat) provide total protein content at levels on par with ABM. However, in order to ensure a balanced amino acid profile, complementation of multiple plant-based proteins is generally necessary. For instance, legume (low in sulfur-containing amino acids, high in lysine) and cereal (low in lysine, high in sulfur-containing amino acids) proteins are favorable complements. Factors that have been identified in plant proteins that may decrease nutrient bioavailability post-ingestion include: structures resistant to proteolysis, protein conformation, and antinutrients (e.g., tannins, phytates, lectins)^45^. Certain processing techniques (e.g., soaking, heating, sprouting) have been shown to increase digestibility^15^. Nutrition is also variable between traditional and novel PBM products. For example, tofu (traditional PBM) and Impossible™ (novel PBM) share certain benefits over ABMs such as containing dietary fiber and minerals while lacking cholesterol. However, tofu-specific benefits include fewer calories, less fat and sodium-free and Impossible™-specific benefits include higher protein and vitamin B_12_ content (Fig. 4). Concern has been expressed regarding the inclusion of LegH in PBM, citing correlations between heme iron intake and increased risk of diabetes^46^.

Comprehensive, baseline nutrition data for CBM is not publicly available. Using small sample sizes, the nutrient content of cell cultures can be quantified via laboratory assays^27^. Different cell types contribute different sets of nutrients; differentiated muscle cells will likely be the primary source of protein and mature adipocytes can contribute to the fatty acid profile^41^. Certain compounds that are provided by ABM are not present in cultured cells. For example, vitamin B_12_ is only synthesized by bacteria and will need to be supplemented^47^. As with flavor, proponents of CBM often claim its nutrition profile will be comparable with or superior to ABM and that nutrients can be tuned via co-cultures, media supplementation, and genetic modification^14^. Media formulation will have a large impact on the viability and efficiency of the cultured cells, on the nutrition profile, and perhaps also impact flavor and taste. Genetic modification for nutritional improvement is another approach that may be more efficient in the long-term, although genetic approaches may pose problems for regulatory strategy and consumer acceptance. Genetic engineering has already been implemented in livestock to improve various aspects of meat production. In 2004, transgenic swine were generated to express a gene originating in spinach with a goal of improving the fatty acid profile of pork^48^. This and other modifications could be implemented on a cellular level to influence the properties of CBM. Comprehensive nutrition data for CBM should become available with the launch of initial products, scale-up, and additional interest from the scientific community.

## Consumer acceptance

Consumer acceptance is of particular interest to PBM stakeholders who are looking to increase market share. A high consumer acceptance for PBM products was recorded in China (95.6%) and India (94.5%), compared to the United States (74.7%)^49^. In a European study, the main barriers for dietary inclusion of PBMs were lack of familiarity and low “sensory attractiveness”, and consumers who were unfamiliar with analog products were more likely to want these products to closely imitate ABM^50^. In a focus group study, motivating factors for not eating ABM ranked differently in Germany (e.g., animal welfare, health, environmental impacts), the Netherlands (e.g., animal welfare, poor meat quality, health), and France (e.g., health, animal welfare, sustainability)^51^. For all three nations, the taste was the key factor inhibiting consumption of plant proteins, with other factors including habit, convenience and price. In a sensory panel study comparing animal-, plant- and insect-based burgers, animal burgers were associated with the emotional terms of ‘contented, happy and pleasant’, while plant burgers were associated with ‘disappointed, distrust and discontented’^52^. Beyond and Impossible™ products introduce a new class of PBM products that more closely mimic ABM compared to the previously established texturized vegetable protein items. These products may be viewed as “highly processed” compared to traditional vegetable burgers and may alienate “clean label” consumers; who are concerned about “unnatural” methods of food production^53^. New consumer acceptance research is required to determine how these products measure up to the findings reported for more established products.

A general consensus in the field is that CBM is targeted at consumers who currently eat meat; as plant-based diets are agreed upon as healthful and sustainable for vegetarian-leaning individuals. Interestingly, vegetarians and vegans in the United States are both more likely to agree with the potential benefits of CBM but less willing to try it compared to omnivores^54^. A systematic review of cultured meat consumer acceptance studies found the most commonly reported concerns were associated with: unnaturalness, safety, healthiness, taste, texture, and price^55^. A 2017 European consumer study found that lack of naturalness decreased acceptance of cultured meat; even with awareness of potential environmental and animal welfare benefits^56^. Along with this finding, research examining internet comments on United States-based news articles covering cultured meat development found more critical input than approval responses, and a frequent critique was that CBM would be “unnatural” and “unappealing”^57^. A 2018 Switzerland study concluded that informing consumers about the production process did not increase acceptance and that communications that emphasize the final product, rather than the technical processes, would be a more successful strategy^58^. Similarly, a 2020 Netherlands study reported that educating consumers on the personal and societal benefits had a positive impact on consumer acceptance^28^. Nomenclature of meat grown from cell cultures may also have an effect on consumer perspective. When comparing the effects of ‘animal-free, clean, cultured’ and ‘lab-grown’ on a panel of participants, ‘animal-free’ and ‘clean’ incited more positive attitudes compared to ‘lab-grown’^59^. Other common descriptors include ‘artificial’, ‘cell-based’, ‘cultivated’, ‘in vitro’, and ‘synthetic’.

## Public health

Meat is an important source of nutrition, especially in developing countries facing nutrition deficiencies. However, the overconsumption of meat has been linked to a number of health concerns. More than 1.8 million people die each year from ischemic heart disease, a quarter of which is linked with overconsumption of certain meat products^60^. Results from a recent clinical trial administered by the Stanford School of Medicine demonstrated that participants who substituted PBM for ABM over eight weeks exhibited lower risk for cardiovascular disease (e.g., reduced fasting serum trimethylamine-N-oxide levels)^61^. The consumption of PBM follows most nutritional dietary guidelines which recommend to limit intake of red and processed meat^62,63^, and could benefit consumers that desire reductions in blood pressure, body mass index, and cholesterol^64^. Foodborne pathogens found in meat, such as *Escherichia coli, Salmonella*, and *Campylobacter*, result in millions of illnesses each year^65^. Though PBMs are generally not associated with pathogenic disease concerns, non-animal products are capable of causing foodborne illness. A 1999 study screening tofu sold at grocery stores determined that 16% of tested samples were contaminated with coliform bacteria^66^. Plant foods can become contaminated with pathogens via contact with contaminated sources of animal manure, water or other foods. Antibiotics are also used in plant agriculture, but at relatively low levels (in the United States, plant use accounts for only 0.12% of animal agriculture antibiotic use)^67^, therefore, compared with ABM, PBM is less associated with antibiotic-related “drug-resistance” issue.

The commercialization of CBM could impact numerous aspects of public health including foodborne illness, nutrition deficiency, diet-related disease (e.g., colorectal cancer, cardiovascular disease), and infectious disease^6^. The risk of foodborne illness from CBM could be theoretically non-existent, since the sterile conditions required for cell proliferation will prevent contamination with disease-causing pathogens, provided that post-processing and packaging procedures are equally sterile. While sterile cell culture is implemented in pharmaceutical manufacturing, it may not be economically feasible for food production. Natural, food-grade antimicrobial agents may be a promising strategy to reduce contamination risk while remaining cost-effective^68^. Similarly, the threat of zoonotic disease transmission could be directly reduced by decreasing human contact with infected animals and indirectly by reducing habitat destruction^69^. Nutrition deficiency and diet-related disease could be addressed with cell selection, genetic modification, and medium formulation strategies to regulate the presence of healthful and unhealthful compounds. Conversely, consumers report concerns with the safety and healthfulness of CBM, citing fears about unnaturalness, cancer, and inadequate regulation^55^. So far, public health claims on both sides are entirely speculative as the relevant research has yet to be published.

## Environmental sustainability

**Fig. 5:**
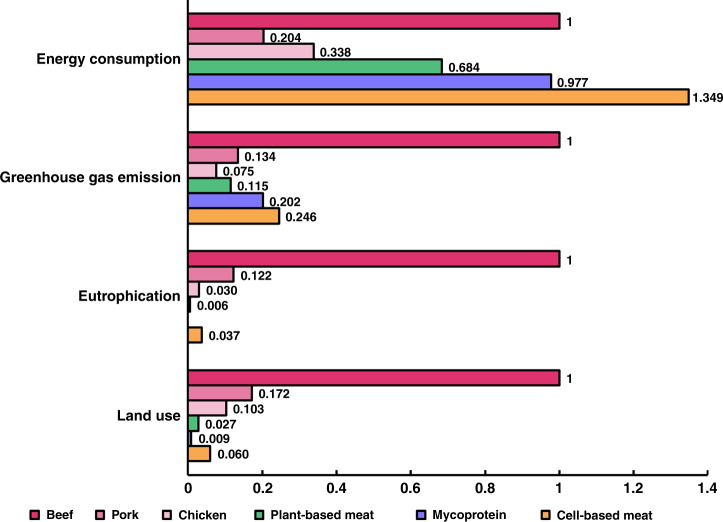
Comparison of the environmental impact of meat and meat analogs. Data are normalized to the impact of beef production. Eutrophication does not include data for mycoprotein. Land, emissions and energy data for mycoprotein were adapted from a 2015 LCA^72^. Data for beef, pork, chicken and CBM were adapted from a 2015 life cycle assessment^75^. Data for PBM were adapted from an Impossible™ Beef LCA (land, eutrophication, emissions) and a Beyond Meat^®^ life cycle assessment (energy use)^70,71^.

Beyond Meat^*®*^ and Impossible™ Foods have both released life cycle assessments (LCA) for their plant-based beef products^70,71^. Eutrophication potential and land use requirements for these products are projected to be significantly lower than metrics reported for factory-farmed animal-based beef, pork and chicken while greenhouse gas emissions fall between metrics for pork and chicken and energy consumption exceeds that of pork and chicken (Fig. 5). Compared to estimates for Beyond and Impossible™, mycoprotein (i.e., Quorn™) is more impactful with regard to energy and emissions but requires less land for production (Fig. 5). The water footprint of PBMs is highly dependent on the source of the main protein. A LCA comparing meat alternatives calculated that mycoprotein-based products (40 kg/kg) have higher water requirements compared to gluten (0.954 kg/kg) and soy-based (0.73 kg/kg) items^72^. A separate LCA study estimated the water usage for 39 distinct meat analogs and determined that, on average, a ton of PBM product consumed 3800 m^3^ of water^73^. A majority of the consumption is due to the processing of meat analogs after harvest of the raw protein sources (transportation and packaging were other factors).

The production of CBM is anticipated to, once optimized, require fewer resources and emit less waste relative to ABM^14^. Anticipated reductions are based on assumptions of: (1) targeted tissue cultivation (i.e., reduced by-products, non-meat tissues); (2) higher production rates and (3) vertical production systems^14^. The first relevant LCA published in 2011 estimated CBM would involve lower energy consumption (7–45%), greenhouse gas emissions (78–96%), land use (99%), and water use (82–96%) compared to ABM^74^. A separate 2015 LCA of CBM reported less dramatic footprint reductions and determined that the energy consumption, acidification potential, and ozone depletion potential impacts of CBM could be more detrimental than ABM, especially when compared to poultry production^75^. CBM is estimated to have a 47% energy feed conversion efficiency and 72% protein feed conversion efficiency, values that are lower than PBM and insect-based meat but higher than ABM^76^.

## Animal welfare

PBM products are generally free of animal byproducts and thus do not have direct negative impacts on animal welfare. However, a subset of products contains dairy-based or egg-based additives and thus are vegetarian but not vegan. Similar to the meat industry, egg, and dairy production methods are major sources of animal welfare concerns. In the egg industry, millions of male chicks that are not suitable for egg production or meat production, are killed each year and the beaks of female birds are trimmed to prevent pecking^77^. In the dairy industry, dairy cows are repeatedly impregnated for continuous milk production and are routinely separated from their calves, which are transported to other farms for veal production; causing extreme emotional distress^78^. Even vegan PBM can have indirect effects on wild animal welfare in the form of habitat destruction. To meet food demands, natural vegetation is cleared with monocultural crops which impacts biodiversity. In 1994, palm oil cultivation in Malaysia was found responsible for decreasing mammal inhabitants from 75 to 10 species per hectare^79^. While all agribusiness has an impact on animal welfare that are worthy of concern, substitution of ABM with PBM is still a substantial improvement for animal welfare, as it avoids the unethical treatment of animals during rearing, transportation and slaughter^80^.

One of the primary proposed benefits of CBM is the improvement of farm animal welfare via supplanting intensive animal agriculture. Animal donors are used to supply initial sources of cells that are subsequently expanded in vitro, without needing further resources from the animal. Donor animals, usually younger animals that have more proliferative cells, are anesthetized by a veterinarian and a small (<1 g) tissue biopsy is removed. Cells could be genetically immortalized to proliferate indefinitely; eradicating the need for animal donors. However, in practice, animal donors will likely be relied upon to maintain genetic diversity and to supply non-genetically modified options for CBM. Aside from cell sourcing, a key aspect of CBM production that is linked to animals is serum supplementation. Fetal bovine serum is a common additive to cell culture media and it provides essential growth factors for mammalian cell culture. In 2003, it was estimated that blood from 1,000,000 bovine fetuses was harvested to generate the annual production of 500,000 liters of fetal bovine serum^81^. Serum-free alternatives include co-culture approaches or supplementation with recombinant growth factors.

## Looking forward

Animal-based meat production has evolved over thousands of years to supply the demand for affordable and appetizing food. Unfortunately, this feat is accompanied with unintended consequences for human health, natural resources, and the animals involved. Driven by both the rise in global meat demand and increased concern about the aforementioned negative externalities, researchers, and entrepreneurs are turning their focus towards animal-free approaches of meat production (Box 1). The economic opportunity for meat alternatives is large and there is no need to crown a single front-runner technology to monopolize the market. Instead, it is important to pursue multiple solutions simultaneously to provide a range of products to serve disparate segments of the consumer market. Plant-based and cell-based meat technologies have made significant advances since their conception. PBM has evolved from being a lackluster meat alternative, that provides a nutritional but not sensory replacement to meat, to be a novel analog nearly indistinguishable from the ABM it seeks to emulate. Likewise, CBM has matured from being the musing of science fiction to being a tangible prototype.

PBM products lie on a spectrum where one end houses more “natural,” less-processed proteins that fall in line with “clean-label” viewpoints but do not do well to mirror the experience of eating meat, while the other contains sensory equivalents that require source proteins to be entirely transformed, and thus viewed as highly processed, and which may come at the cost of certain nutritional factors. Mycelium-based meat may be an exception, where biofermentation can be employed to utilize natural structures and growth patterns of filamentous fungi to mimic meat structures. Along these lines, screening new protein sources that exist in nature that may emulate meat without excessive human manipulation may be an approach that appeals to a wider pool of consumers. CBM is impeded by high production costs, scale-up hurdles, and gaps in fundamental knowledge surrounding how to employ cell culture for food applications. In particular, there are no peer-reviewed, comprehensive datasets detailing the cost, sensory properties, or nutritional value of cell-cultured tissues. While published assessments of environmental impact projections exist, they are based on theoretical large-scale processes that have yet to be validated by industry. To that point, it will be necessary to ascertain details, such as the parameters of material inputs (e.g., doubling time, maximum cell density, medium composition) and industrial-scale production schematics (e.g., bioreactor design, operations) before cost, impact and food safety can be reliably analyzed.

There are opportunities for plant-based and cell-based hybrid products. Considering the current high-cost hurdles associated with CBM, one approach is to focus on the aspects of PBM that fall short of ABM and determine where CBM can add the most value at the lowest inclusion rate. For example, combining PBM with cell-cultured fat may improve the sensory properties of the analog while remaining less costly than a pure CBM product. To this end, expanding research goals to answer fundamental questions surrounding the cost, sensory and nutrition profiles is important to further inform stakeholders on the best areas of application for CBM. Finally, eventual success of PBM and CBM in the marketplace could transform, rather than eliminate, ABM production. If demand for lower quality, previously factory-farmed meat can be supplied with PBM and CBM, demand for higher quality, ABM could be met by smaller-scale, more sustainable and more humane methods of animal farming.

Box 1. A summary of key comparisons between animal-based, plant and fungi-based and cell-based approaches to meat production **History & Approach** Meat consumption has been commonplace since the beginning of human evolution, while plant-based meat analogs are relatively recent dietary additions. Cell-based meat products have not been commercialized, but people have taste-tested prototypes produced by companies and via a singular academic study. Approaches to meat production can be primarily differentiated by starting material: animals (ABM), plants or fungi (PBM) or cells (CBM).

**Economics** Advances in farming technology and intensification of animal agriculture have resulted in inexpensive and accessible ABM. Although raw inputs for PBM (i.e., unprocessed plant proteins) are less costly than for ABM (i.e., livestock), PBM retail prices are consistently higher due to costs associated with post-processing, production scale and supply chains. Current CBM production is not commercially feasible. Increases in production scale and culture medium-cost reductions are necessary to improve the economics of the process.

**Regulatory framework** Regulation of novel foods such as certain PBM products and all CBM products is a current topic of interest. Namely, there is a lack of precedent for oversight of food containing cultured cells. Novel additives for PBM may fall within existing regulatory frameworks (e.g., Novel Food Regulation (Europe), “Generally Recognized as Safe” (United States) pathways). There are ongoing debates surrounding labeling laws of what products can be characterized as meat.

**Organoleptic properties** The primary sensory properties of meat are appearance, aroma, flavor, and texture. Next-generation PBM products are increasingly successful at mimicking processed ABM products (e.g., burgers, deli meat). Current strategies for producing structured PBM products (e.g., filets, steaks) include extrusion, shear cell technology, 3D printing, and mycelium cultivation. Early CBM prototypes appear to emulate ABM but few people have been able to taste-test these samples. The first published sensory panel feedback of a CBM prototype (i.e., cow cells on a texturized soy protein scaffold) reported “a pleasant meaty flavor” and “a typical meat bite and texture”.

**Nutrition** ABM is a good source of essential amino acids, minerals and vitamins. Some nutritional benefits of traditional PBMs include an absence of cholesterol while providing sources of dietary fiber and healthy fatty acids. Improvements in organoleptic properties may come at the cost of certain nutritional aspects. For example, notable novel PBM products contain high sodium content. There are no publicly available datasets regarding the nutritional profile of CBM. Proponents of the technology assert that nutrition can be regulated by adjusting culture medium formulations and implementing co-culture strategies or genetic modifications.

**Consumer acceptance** The motivations behind consumption of PBM vary depending on consumer nationality and China and India report higher consumer acceptance rates of PBM compared to the United States. Next-generation PBM products can be perceived as “highly processed” and may not be appealing to “clean label” consumers. With regard to CBM, plant-based consumers are more likely to agree with CBM’s proposed benefits but are less willing to try it compared to omnivores. Consumer perception of CBM is influenced by marketing focus (e.g., process vs. product vs. impact) and terminology (e.g., lab-grown vs. clean vs. cultured).

**Public health** Overconsumption of red and processed meat is linked to a number of health concerns. There is some evidence that substituting PBM analogs for ABM products can decrease risk factors associated with cardiovascular disease. Compared to ABM, the production of PBM is less associated with pathogenic disease and antibiotic resistance issues. The impact of CBM on public health issues depends on how large-scale production schemes evolve (e.g., sterile processes, antibiotic use). Some consumers report concerns about the safety of CBM products, citing apprehension about unnaturalness and inadequate regulations.

**Environmental sustainability** Life cycle analyses conclude PBM products are generally more environmentally sustainable than animal-based beef. Some metrics (e.g., greenhouse gas emissions, energy consumption) for some novel PBM products are less sustainable when compared to animal-based poultry. PBM water footprints vary widely depending on the main protein source. The environmental impact of CBM is highly debated, with preliminary assessments presenting significant degrees of uncertainty; especially for energy consumption values.

**Animal Welfare** Billions of animals suffer and die each year as a direct result of ABM production. Vegan PBM products do not have as direct impacts on animal welfare but crop cultivation can contribute to the destruction of wildlife habitats. One of the primary proposed benefits of CBM is the improvement of farm animal welfare. However, CBM production currently utilizes donor animals for cell acquisition and the culture medium is composed of animal-derived components. The creation of immortalized cell lines and animal-free culture medium ingredients are proposed to address these issues.
